# The role of chemoprevention by selective cyclooxygenase-2 inhibitors in colorectal cancer patients - a population-based study

**DOI:** 10.1186/1471-2407-12-582

**Published:** 2012-12-06

**Authors:** Yi-Hsin Yang, Yea-Huei Kao Yang, Ching-Lan Cheng, Pei-Shan Ho, Ying-Chin Ko

**Affiliations:** 1 School of Pharmacy, College of Pharmacy, Kaohsiung Medical University, Kaohsiung, Taiwan; 2 Cancer Center, Kaohsiung Medical University Hospital, Kaohsiung, Taiwan; 3 Institute of Clinical Pharmacy and Pharmaceutical Sciences, Health Outcome Research Center, National Cheng Kung University, Tainan, Taiwan; 4 Department of Oral Hygiene, College of Dental Medicine, Kaohsiung Medical University, Kaohsiung, Taiwan; 5 Center of Excellence for Environmental Medicine, Kaohsiung Medical University, Kaohsiung, Taiwan; 6 Graduate Institute of Clinical Medical Science, China Medical University, Taichung, Taiwan; 7 Enviroment-Omics-Disease Reserach Center, China Medical University Hospital, Taichung, Taiwan

**Keywords:** Chemoprevention, Colorectal cancer, Selective COX-2 inhibitor, Population-based study

## Abstract

**Background:**

There are limited population-based studies focusing on the chemopreventive effects of selective cyclooxygenase-2 (COX-2) inhibitors against colorectal cancer. The purpose of this study is to assess the trends and dose–response effects of various medication possession ratios (MPR) of selective COX-2 inhibitor used for chemoprevention of colorectal cancer.

**Methods:**

A population-based case–control study was conducted using the Taiwan Health Insurance Research Database (NHIRD). The study comprised 21,460 colorectal cancer patients and 79,331 controls. The conditional logistic regression was applied to estimate the odds ratios (ORs) for COX-2 inhibitors used for several durations (5 years, 3 years, 1 year, 6 months and 3 months) prior to the index date.

**Results:**

In patients receiving selective COX-2 inhibitors, the OR was 0.51 (95% CI=0.29~0.90, p=0.021) for an estimated 5-year period in developing colorectal cancer. ORs showing significant protection effects were found in 10% of MPRs for 5-year, 3-year, and 1-year usage. Risk reduction against colorectal cancer by selective COX-2 inhibitors was observed as early as 6 months after usage.

**Conclusion:**

Our results indicate that selective COX-2 inhibitors may reduce the development of colorectal cancer by at least 10% based on the MPRs evaluated. Given the limited number of clinical reports from general populations, our results add to the knowledge of chemopreventive effects of selective COX-2 inhibitors against cancer in individuals at no increased risk of colorectal cancer.

## Background

Colorectal cancer (CRC) is currently a common cancer in many countries [1]. In Taiwan it is the second leading cause of cancer-related death, with a 5-year survival rate of 56% and a median age of 68 years [2]. The incidence of CRC is a global health problem, and the search for chemopreventive agents to inhibit its carcinogenesis is urgently required.

Cyclooxygenase-2 (COX-2) has been found to be over-expressed in a number of cancers, including CRC, and has been shown to stimulate tumorigenic pathways [3,4]. Therefore, COX-2 is a valid target for inhibiting or preventing carcinogenesis [3,5]. Non-steroidal anti-inflammatory drugs (NSAIDs) inhibit both isoforms of cyclooxygenase (COX-1 and COX-2). In the gastrointestinal tract, COX-1 produces prostanoids that are involved in the defense and repair of the gastrointestinal mucosa, while COX-2 is expressed in response to inflammatory stimulation [3]. Variation in the chemical structure of existing NSAIDs results in different specificities for COX-1 and COX-2 [6]. Traditional NSAIDs, such as aspirin, are generally less selective for COX-2, whereas Coxibs (celecoxib, rofecoxib) have higher COX-2 selectivity. Given the different roles of COX enzymes in the gastrointestinal tract, selective COX-2 inhibitors have been shown to have less gastrointestinal toxicity than traditional NSAIDs [4].

Most clinical studies investigating the chemopreventive role of selective COX-2 inhibitors have been conducted in Western populations [7]. Therefore, it is of interest to conduct similar population-based studies in an Asian population so that comparisons among demographic groups can be made. The Taiwan Health Insurance Research Database (NHIRD) contains all health insurance claims made in the Taiwanese population, serving as a useful resource to conduct this type of population-based study.

The purpose of this study is to assess the trends and dose–response effects of various medication possession ratios (MPR) for selective COX-2 inhibitor usage in chemoprevention of CRC. Furthermore, subgroups of gender and age categories are compared.

## Methods

### Data source

The National Health Insurance (NHI) program was initiated in 1995 and covers all medical services in Taiwan. The coverage of the NHI program was initially 93.1% of the entire Taiwanese population in 1996, rising to 99.6% by 2010. The program’s National Health Insurance Research Database (NHIRD) contains inpatient and outpatient medical and prescription drug claims as well as the demographic data of all beneficiaries. We used two sets of data from the NHIRD in this study to construct our case and control groups. This ethics of using the database and the study design was reviewed and approved by the Institutional Review Board of Kaohsiung Medical University Hospital (KMUH-IRB-980174).

### Case group

We retrieved an 11-year longitudinal database (1997–2007) of patients who have at least one diagnosis of ICD 9 (International Classification of Diseases revision 9 code 140–208) from the NHIRD. This database includes records of inpatients, outpatients and pharmaceuticals. As these patients were reported in the NHI database for cancer screening purposes, the actual CRC patients could be identified by linking their encrypted personal identification number to the Registry for Catastrophic Illness patients with ICD 9 code 153–154. The date of first diagnosis was considered the index date for each patient.

For the period 2002–2006, we identified 42,358 CRC patients from the database. For the same period, the number of cancer cases reported by the Taiwan Cancer Registry was 46,432 across all ages [2]. Thus, the patients we identified accounted for 91% of the total Cancer Registry patients. We excluded patients whose age was not between 18 and 100 years old or who were diagnosed with other cancers (ICD 9 code 140–208, except 153–154) or benign lesions (ICD 9 code 210–239) prior to the index date.

### Control

We selected controls from the Longitudinal Health Insurance Database 2005 (LHID2005, years 1996–2006). The LHID2005 contains all the original claims of 1,000,000 beneficiaries, randomly sampled from the Registry for Beneficiaries (ID) of the NHI database in 2005. According to the NHIRD report, there was no significant demographical difference between the patients in the LHID2005 and the whole National Health Insurance database. At most, 10 randomly selected controls, without any history of cancer (ICD9 code 140–208) or benign neoplasm (ICD9 code 210–239), were matched with each CRC patient in terms of gender and birth year. The index date of each CRC patient was assigned as the index date to each of the matched controls.

### Drug categories and dosage

The selectivity of a given NSAID can be expressed by the COX-1/COX-2 IC50 ratio. Drugs which are more selective for COX-2, such as coxibs, have lower IC50 ratios than traditional NSAIDs [8]. Selective COX-2 inhibitors (celecoxib and rofecoxib) became eligible for reimbursement by the NHI program starting in 2001. Rofecoxib was withdrawn from the market in 2004, therefore celecoxib is the only currently recorded selective COX-2 inhibitor in the NHIRD. In addition to selective COX-2 inhibitors, we used data from patients using traditional NSAIDs (indomethacin, sulindac, diclofenac, acemetacin, ketorolac, piroxicam, ibuprofen, naproxen, ketoprofen and mefenamic acid) and preferential COX-2 inhibitors (nabumetone, meloxicam, etodolac and nimesulide) as covariates in statistical analyses.

We determined patient usage of the three prescribed drug types (selective COX-2 inhibitors, traditional NSAIDs and preferential COX-2 inhibitors) from data obtained by the Details of Inpatient Orders (DO) and Details of Ambulatory Care Orders (OO) from the Original Claim Database. Information obtained included delivery dates, number of tablets, capsules or other dispensation vehicles, drug dosage, and duration of the prescription period. We used all prescriptions of oral traditional NSAIDs, selective COX-2 inhibitors and preferential COX-2 inhibitors filled during the follow-up period as independent variables in our statistical analyses. The defined daily dosage (DDD), which is the average dosage of a drug taken by adults for the most frequent indication, was computed according to the anatomic therapeutic chemical (ATC) classification system from WHO [9].

### Follow-up groups

**Figure 1:**
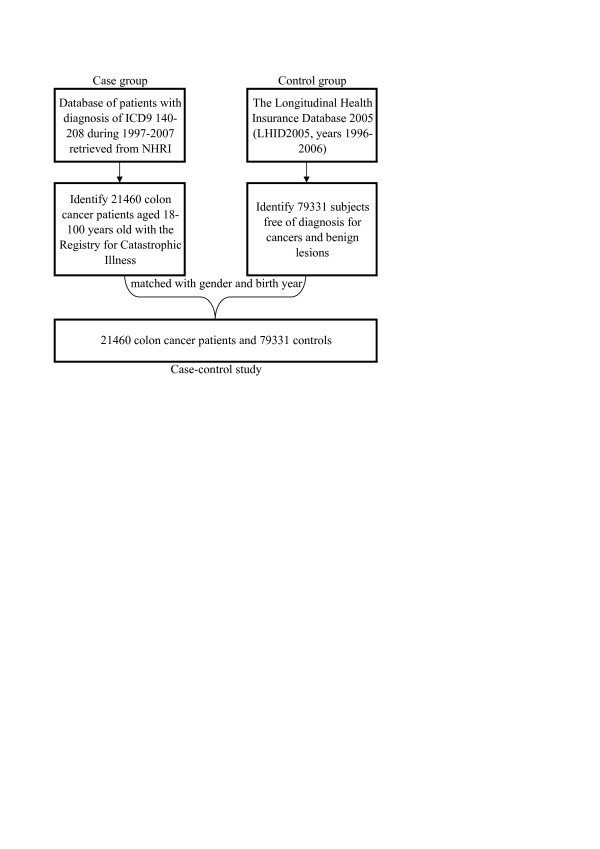
Flowchart of data acquisition.

We created three follow-up groups of different durations (all beginning in 1997) to ensure each patient had the same observation period, and to maximize the number of subjects for our analysis. The 5-year follow-up group had patients with full 5-year observation records before their index date, and hence, only cancer patients with their first diagnosis between 2002 and 2006 were included (Figure 1). Similarly, for the 3-year and 1-year follow-up groups, cancer patients with their first diagnosis between 2000 and 2006 and between 1998 and 2006, respectively, were included. We used the 1-year follow-up group to obtain data regarding patients that used the drugs for 3 and 6 months.

### Statistical analysis

Each CRC patient and the corresponding matched controls were considered as a stratum in the matched case–control study. During variable analysis between cases and controls, each case was matched with 10 controls. We used the reciprocals of the values in the control group and applied them as weights in the estimates and hypothesis testing.

We used conditional logistic regression to determine the estimated drug effects, as determined by their odds ratios (ORs) and 95% confidence intervals (CI), of selective COX-2 inhibitors used over different durations (5 years, 3 years, 1 year, 6 months and 3 months). The medication possession ratios (MPRs) of the inhibitors, calculated by dividing the cumulative DDD by the total number of days in each follow-up period, were used as a continuous independent variable.

The MPRs of selective COX-2 inhibitors were ordered in increments of 10% with 10% and 90% as cut-off points. For subjects with an MPR of 10%, 50%, or 90%, we generated three categorical variables for each: subjects taking the drug for at least 50% of their follow-up period; subjects taking the drug for less than 50% of the time; and non-users (the reference group). In total, nine separate conditional logistic models were generated for these three MPRs.

In addition to the variables calculated for selective COX-2 inhibitors, we added the following covariates into the conditional logistic regression analysis models: 1) MPRs of traditional NSAIDs and preferential COX-2 inhibitors; 2) three categories of insured payroll claims; 3) five different residential areas; and 4) comorbidities with dichotomous variables for 15 medical conditions. We used the ICD 9 codes specified in the Charlson comorbidity index [10,11] for the 15 diseases were used to define diseases that were present within the same duration of cumulative DDD before the index dates. Any recorded diagnosis in inpatient or outpatient records would be considered as having diseases.

### Sensitivity analysis

Sensitivity analyses were conducted in this study with a series of 5-, 3- and 1-year follow-up groups. The use of selective COX-2 inhibitors can have various side effects, including congestive heart failure or cardiovascular disorders. We also conducted separate analyses on participants without any occurrence of myocardial infarction or congestive heart failure, without any occurrence of peptic ulcer disease, and without any occurrence of colon or rectal polyps. For patients with occurrence of diseases, only patients with peptic ulcer disease had sufficient sample size for conditional logistic regressions.

## Results

The study database of the 5-year follow-up group comprised 21,460 cases and 79,331 controls. The basic characteristics for the 5-year follow-up groups are shown in Table 1. At the index dates, the average (±sd, standard deviation) age of subjects in the group of 65–100 year olds was 75.20 (±6.67) years, and in the group of 18–64 year olds it was 52.45 (±9.21) years. Characteristics regarding basic information and potential confounding variables are given in Table 1. For prevalence rates of comorbidity, the prior 5-year prevalence rates of congestive heart failure (8.7% vs. 7.5%), peptic ulcer disease (37.2% vs. 27.6%), mild liver disease (19.1% vs. 15.5%), diabetes (22.6% vs. 19.0%) and renal disease (8.5% vs. 7.2%) were significantly higher in the CRC group, and the prevalence of dementia was higher in the control group. The proportions of having at least one prescription in the prior 5 years for selective COX-2 inhibitors were not significantly different (p=0.595); however, the average cumulative defined daily dose (DDD) differed significantly between CRC patients and controls (78.0±151.1 vs. 85.5±120.5, p=0.010).

**Table 1:** Basic characteristics among patients and controls

		**Cancer patients**		**Controls**		
**Variable**	**Item**	**n**	**%**	**n**^**a**^	**%**^**a**^	**p-value**
total		21460		79331		
sex						
	male	12882	60.0	47621	60.0	
	female	8578	40.0	31710	40.0	
age group						
	18-64 years old	9072	42.3	33536	42.3	
	65-100 years old	12388	57.7	45795	57.7	
income category						
	not working	6958	32.4	23361	29.4	<.001
	monthly income<= NT$20000	11019	51.3	45805	57.7	
	monthly income> NT$20000	3483	16.2	10165	12.8	
comorbidity in 5 years before index date						
	Myocardial infarction	486	2.3	1784	2.2	0.888
	Congestive heart failure	1864	8.7	5917	7.5	<.001
	Peripheral vascular disease	609	2.8	2315	2.9	0.534
	Cerebrovascular disease	3724	17.4	13514	17.0	0.273
	Dementia	655	3.1	2825	3.6	<.001
	Chronic pulmonary disease	7002	32.6	25707	32.4	0.534
	Rheumatologic disease	692	3.2	2552	3.2	0.957
	Peptic ulcer disease	7974	37.2	21875	27.6	<.001
	Mild liver disease	4092	19.1	12302	15.5	<.001
	Diabetes	4853	22.6	15049	19.0	<.001
	Diabetes with chronic complications	1462	6.8	4589	5.8	<.001
	Hemiplegia or paraplegia	416	1.9	1609	2.0	0.408
	Renal disease	1833	8.5	5734	7.2	<.001
	Moderate or severe liver disease	94	0.4	298	0.4	0.191
selective COX-2 inhibitors						
	No	18872	87.9	69896	88.1	0.504
	Yes	2588	12.1	9435	11.9	
	Average (±sd) DDD	78.0 (±151.1)		85.5 (±231.7)		0.014^b^
tNSAID						
	No	2014	9.4	8517	10.7	<.001
	Yes	19446	90.6	70814	89.3	
	Average (±sd) DDD	87.2 (±145.8)		94.5 (±170.0)		0.001^b^
preferential COX-2 inhibitors						
	No	15681	73.1	57451	72.4	0.058
	Yes	5779	26.9	21880	27.6	
	Average (±sd) DDD	61.6 (±121.9)		62.2 (144.6)		0.278^b^

a: weighted according to the matched sizes of cases.

b: Two-sample t-tests were conducted at the natural logarithm of average DDD.

DDD: define daily dose; NT: new taiwan dollar; sd: standard deviation; tNSAID: traditional NSAID.

The estimated effects (odds ratios, ORs) of drug usages in various durations (5-year, 3-year, 1 year, 6 months and 3 months) prior to the index dates were investigated by separate conditional logistic regressions with MPR of selective COX-2 inhibitors as a continuous independent variable together with covariates (Table 2). The analyses were conducted in the total subject group and also subgroups of age (age>=65, age<65) and gender (males, females). It was estimated that for people taking selective COX-2 inhibitors for the whole 5 years prior to the index date the OR was 0.51 (95% CI=0.29~0.90, p=0.021) for developing CRC, and the OR was smaller (0.36, 95% CI=0.08~1.67, p=0.193) in people aged less than 65, and was larger (0.57, 95% CI=0.31~1.07, p=0.079) in people aged 65 years old or older. The comparison of estimated ORs between males and females was similar (males: OR=0.48, 95% CI=0.19~1.24, p=0.131; females: OR=0.52, 95% CI=0.25~1.08, p=0.080).

**Table 2:** Estimated odds ratios for taking selective COX-2 Inhibitors during various prior duration

**Duration prior to index date**	**Colon cancer patients**		**Controls**		**Odds Ratio**	**95% confidence intervals**	**p-value**
	**# with at least 1 prescription**	**# of nonusers**	**# with at lease 1 prescription**	**# of nonusers**			
all subjects aged 18-100 years old							
5 years	2588	18872	9435	69896	0.51	(0.29, 0.90)	0.021
3 years	2393	25117	8293	90607	0.58	(0.39, 0.86)	0.007
1year	1264	31959	4153	121708	0.60	(0.46, 0.80)	<.001
6 months	822	35401	2619	123242	0.72	(0.56, 0.93)	0.012
3 months	533	35690	1652	124209	0.80	(0.64, 1.01)	0.056
subjects with aged 65-100 years old							
5 years	2243	10145	8242	37553	0.57	(0.31, 1.07)	0.079
3 years	2073	13600	7247	49098	0.63	(0.41, 0.97)	0.035
1year	1092	19000	3630	66182	0.64	(0.48, 0.88)	0.005
6 months	706	19386	2282	67530	0.72	(0.55, 0.95)	0.021
3 months	461	19631	1450	68362	0.83	(0.65, 1.07)	0.147
subjects with aged 18-64 years old							
5 years	345	8727	1193	32344	0.36	(0.08, 1.67)	0.193
3 years	320	11517	1046	41508	0.45	(0.15, 1.34)	0.152
1year	172	15959	524	55525	0.53	(0.25, 1.11)	0.090
6 months	116	16015	336	55713	0.82	(0.46, 1.48)	0.520
3 months	72	16059	201	55848	0.73	(0.43, 1.26)	0.262
male subjects							
5 years	1185	11697	4175	43446	0.48	(0.19, 1.24)	0.131
3 years	1085	15145	3673	54675	0.57	(0.30, 1.08)	0.085
1year	563	20298	1799	70685	0.59	(0.38, 1.91)	0.016
6 months	366	20495	1095	71389	0.70	(0.47, 1.05)	0.087
3 months	237	20624	660	71824	0.80	(0.56, 1.13)	0.203
female subjects							
5 years	1403	7175	5260	26450	0.52	(0.25, 1.08)	0.080
3 years	1308	9972	4621	35931	0.59	(0.35, 0.98)	0.042
1year	701	14661	2354	51023	0.61	(0.42, 0.89)	0.009
6 months	456	14906	1523	51854	0.73	(0.52, 1.01)	0.056
3 months	296	15066	992	52385	0.80	(0.60, 1.07)	0.136

When considering different duration of selective COX-2 inhibitor usages prior to the index date, the ORs increased from 0.51 (95% CI=0.29~0.90, p=0.021) of 5 year-usage to 0.80 (95% CI=0.64~1.01, p=0.056) of 3 month-usage. Significant differences appeared with the 6-month, 1-year, 3-year and 5-year usages. For the older age group (age>=65 years old), the ORs increased from 0.57 (95% CI=0.31~1.07, p=0.079) for 5 year-usage to 0.83 (95% CI=0.65~1.07, p=0.147) for 3 month-usage. Only the usages of 3-years, 1-year and 6-months were shown to be statistically significant. Although the younger age group (aged 18–64) had smaller ORs from 0.36 (95% CI=0.08~1.67, p=0.193) for 5 year-usage to 0.73 (95% CI=0.43~1.26, p=0.262) for 3 month-usage, none of these estimated effects were significant. The comparison of estimated ORs between males and females was also similar. Significant drug usage effects were found at 1-year usage by males (OR=0.59, 95% CI=0.38~0.91, p=0.016) and at 3-year and 1-year usage by females (3-year: OR=0.59, 95% CI=0.35~0.98, p=0.042; 1-year: OR=0.61, 95% CI=0.42~0.89, p=0.009).

**Figure 2:**
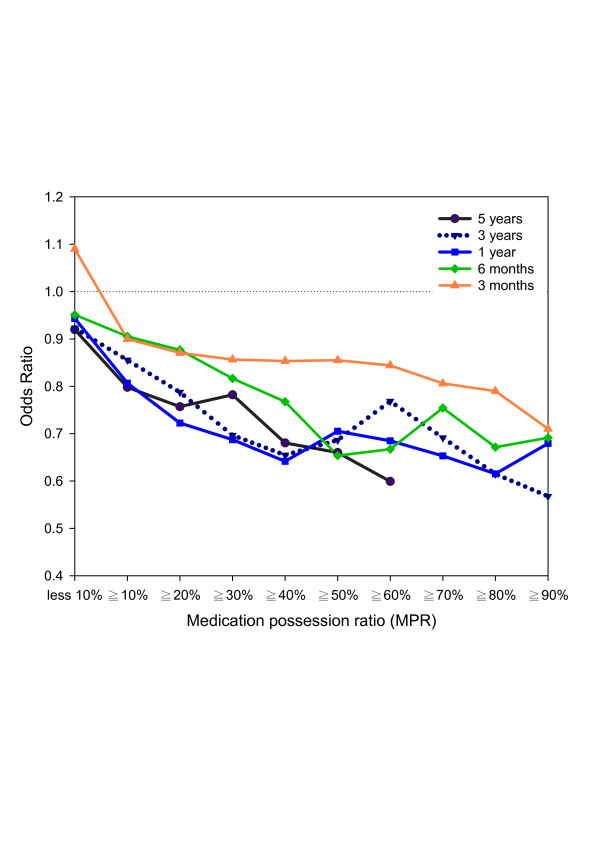
**Odds ratios for developing colorectal cancer in different MPRs for selective COX-2 inhibitors.** (Note: Significant odds ratios are at 10% and 20% of 5-year cumulative usage, at 10% to 40% of 3-year usage, at 10% to 80% of 1-year usage, and at 30% to 60% and 80% of 6-month usage).

**Figure 3:**
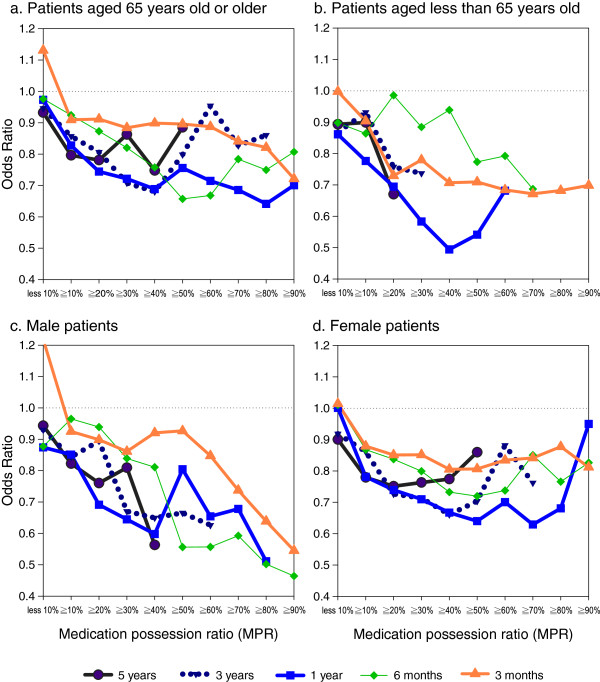
Odds ratios for developing colorectal cancer in different MPRs for selective COX-2 inhibitors in (a) age>=65, (b) age<65, (c) males and (d) females (Note: Significant odds ratios are at (a) 10% of 5-year, 10% to 40% of 3-year, 10% to 50% of 1-year, 30% to 60% of 6-month, and 90% of 3-month; (d) 10% of 5-year, 20% to 40% of 3-year, 10% to 50% of 1-year, 40% of 6-month).

To investigate the risk reduction at various MPRs of drug usage for prior durations, the estimated ORs were computed by using indicator variables for at least 10% to 90% (in 10% intervals) of MPRs at follow-up durations in 9 separate conditional logistic regressions with “no use” as the reference category. These estimated ORs with standard error of parameter estimate less than 0.45 (equivalent to all cell sizes larger than 5) are plotted in Figure 2. The risk reduction curves, which consist of ORs, decrease as the MPRs increase, and all of the estimated ORs show protection effects (ORs<1). ORs showing significant protection effects are at 10% and 20% of 5-year cumulative usage, at 10% to 40% of 3-year usage, at 10% to 80% of 1-year usage, and at 30% to 60% and 80% of 6-month usage. Except for the 3-month curve, the other 4 curves (5-year, 3-year, 1-year and 6-month) are closer together. Figure 3 shows plots for subgroups of age (age>=65 and age<65) and gender (males and females). The ORs for the least 10% of usage were more heterogeneous in people with age less than 65 and in males.

For the sensitivity analyses (Table 3), using the 5-year follow-up group for the 3-year and 1-year analyses reveals similar estimates only with less statistical significance. When considering participants without any prior history of cardiovascular events, the estimated ORs are not very different.

**Table 3:** Sensitivity analyses

**Sensitivity analysis**	**Colon cancer patients**		**Controls**		**Odds Ratio**	**95% confidence intervals**	**p-value**
	**# with at least 1 prescription**	**# of nonusers**	**# with at least 1 prescription**	**# of nonusers**			
5-year follow-up prior to index date							
main analysis: maximum numbers of participants in different years of follow-up	2588	18872	9435	69896	0.51	(0.29, 0.90)	0.021
using the same 5-year follow-up group for 3- year and 1-year analyses	2588	18872	9435	69896	0.51	(0.29, 0.90)	0.021
participants without any occurance of myocardial infarction or congestive heart failure	2048	17238	7584	64687	0.57	(0.30, 1.08)	0.085
patients without any occurance of peptic ulcer disease	1112	12374	4597	52859	0.61	(0.19, 1.97)	0.412
patients with any occurance of peptic ulcer disease	1476	6498	4838	17037	0.31	(0.08, 1.19)	0.089
patients without any occurance of colon or rectal polyps	2520	18447	9432	69895	0.55	(0.30, 1.98)	0.044
3-year follow-up prior to index date							
main analysis: maximum numbers of participants in different years of follow-up	2393	25117	8293	90607	0.58	(0.39, 1.86)	0.007
using the same 5-year follow-up group for 3- year analysis	2376	19084	8489	70842	0.60	(0.40, 0.89)	0.011
participants without any occurance of myocardial infarction or congestive heart failure	1990	23458	7001	85573	0.61	(0.39, 0.94)	0.026
patients without any occurance of peptic ulcer disease	1190	17857	4737	74165	0.71	(0.37, 1.36)	0.304
patients with any occurance of peptic ulcer disease	1203	7260	3556	16442	0.49	(0.20, 1.20)	0.117
patients without any occurance of colon or rectal polyps	2316	18671	8488	70841	0.61	(0.40, 0.91)	0.016
1-year follow-up prior to index date							
main analysis: maximum numbers of participants in different years of follow-up	1264	31959	4153	121708	0.60	(0.46, 0.80)	<.001
using the same 5-year follow-up group for 3-year and 1-year analyses	1247	20213	4380	74951	0.61	(0.46, 0.81)	0.001
participants without any occurance of myocardial infarction or congestive heart failure	1139	33696	3807	118187	0.59	(0.43, 0.79)	0.001
patients without any occurance of peptic ulcer disease	765	28501	2951	111326	0.79	(0.54, 1.15)	0.227
patients with any occurance of peptic ulcer disease	499	3458	1202	10382	0.56	(0.17, 1.85)	0.340
patients without any occurance of colon or rectal polyps	1216	19797	4379	74951	0.61	(0.46, 0.81)	0.001
6-month follow-up prior to index date							
main analysis: maximum numbers of participants in different years of follow-up	822	35401	2619	123242	0.72	(0.56, 0.93)	0.012
using the same 5-year follow-up group for 3- year and 1-year analyses	806	20654	2747	76584	0.72	(0.56, 0.93)	0.013
participants without any occurance of myocardial infarction or congestive heart failure	744	34091	2411	119583	0.70	(0.53, 0.91)	0.009
patients without any occurance of peptic ulcer disease	491	28775	1855	112422	0.95	(0.68, 1.33)	0.777
patients with any occurance of peptic ulcer disease	331	6626	764	10820	0.67	(0.24, 1.88)	0.443
patients without any occurance of colon or rectal polyps	786	20227	2747	76583	0.71	(0.55, 0.92)	0.009
3-month follow-up prior to index date							
main analysis: maximum numbers of participants in different years of follow-up	533	35690	1652	124209	0.80	(0.64, 1.01)	0.056
using the same 5-year follow-up group for 3-year and 1-year analyses	521	20939	1721	77610	0.80	(0.64, 1.01)	0.057
participants without any occurance of myocardial infarction or congestive heart failure	480	34355	1502	120492	0.78	(0.61, 0.98)	0.036
patients with any occurance of peptic ulcer disease	209	6748	511	11073	0.76	(0.31, 1.85)	0.550
patients without any occurance of colon or rectal polyps	507	20506	1721	77609	0.79	(0.63, 0.99)	0.038

## Discussion

Potential chemopreventive benefits were investigated using a database of cancer patients, and a large database of Taiwanese patients. We were able to demonstrate a dose–response protective effect for selective COX-2 inhibitors, which was related to the occurrence of CRC in individuals.

Based on our results, the proportion of people prescribed at least one COX-2 inhibitor was not significantly different (p = 0.595) between CRC patients and the control group. Usage of selective COX-2 inhibitors between the 2 groups was only different for cumulative DDD. This suggested a potential dose–response relationship for risk reduction. Significant reduction in risk regarding CRC was found for those taking selective COX-2 inhibitors over 6 months (28%, OR = 0.72), 1 year (40%, OR = 0.60), 3 years (42%, OR = 0.58) and 5 years (49%, OR = 0.51). In the group with subjects aged 65 or younger, there was a more pronounced reduction in risk (63% following 5 years of use), however there was no statistical significance. Risk reduction was similar between males and females, and could even be observed at MPRs as low as 10%. In terms of various MPRs (Figure 2), except for the 3-month curve, the other 4 curves (5-year, 3-year, 1-year and 6-month) are closer together. Given that all of the MPRs from 3-month were all not statistically significant, these results might suggest a potential minimum treatment period for chemopreventive effects. In addition, a U-shaped curve can be observed from female patients indicated the protection effects were not associated with increased MPRs. Future studies may look into the disappearance of protection trend by identifying common diseases requiring long-term medication treatment in females.

COX-2 has been found to be over-expressed in many cancers, including CRC [3]. Blockade of COX-2 would down-regulate its metabolic product, PGE2, thereby decreasing the risk of CRC [3]. Prostaglandin levels correlate with disease activity and are consequently correlated with COX expression. This is especially so for COX-2. Prostaglandins derived from COX-1 and COX-2 appear to play a protective role. Theoretically, NSAIDs and COX-2 inhibitors should be capable of inhibiting intestinal production of prostaglandins involved in tissue repair processes. However, previous research has demonstrated conflicting data in animal and clinical studies [3].

Patients administered celecoxib show reduction in size and number of adenomas [1]. Bertagnolli et al. [12] studied patients with prior history of adenomas, and reported that the risk of developing one or more adenomas in 3 years was reduced by 33% in patients treated with 200 mg of celecoxib. Risk was reduced by 45% in patients given 400mg of celecoxib as compared with the placebo group. The Prevention of Colorectal Sporadic Adenomatous Polyps (PreSAP) trial [13] studied similar patients, and showed a 36% reduction in adenoma recurrence and 51% reduction in advanced adenoma in patients taking 400 mg of celecoxib once a day. A meta-analysis [7] of the two clinical trials [12,13] showed a 44% reduction in the recurrence of any adenoma, and a 55% reduction in advanced adenoma during 3 years of follow-up. For rofecoxib, the Adenomatous Polyp Prevention on Vioxx (APPROVe) trial [14] identified that adenoma recurrence was less frequent (RR = 0.76) in the rofecoxib group. Chemopreventive effects were more pronounced in the first year (RR=0.65) than in the subsequent two years (RR=0.81) [3]. From that study, the 3-year risk reduction was estimated at 42% (OR = 0.58, 95% CI = 0.39~0.86), indicating a similar protective effect regardless of CRC occurrence. The median ages of subjects in the two celecoxib studies were 61 [13] and 59 [12] years. In our study, we observed a 55% risk reduction in people younger 65, decreasing further to 37% for those 65 and older.

To date, no reports have been published investigating the chemopreventive roles of non-aspirin NSAIDs, especially selective COX-2 inhibitors, in general populations [7]. An earlier study [15] investigating the effects of aspirin and other NSAIDs on risk reduction revealed an OR of 0.76 (95% CI = 0.58~1.00) for colon cancer, and 0.75 (95% CI = 0.49~1.14) for a 3-year follow-up with at least seven prescriptions.

Selective COX-2 inhibitors are associated with increased risk of cardiovascular events [7,16]. The withdrawal of rofecoxib [14], along with the early termination of the Bertagnolli [12] and Arber [13] studies were all because of more serious adverse cardiovascular adverse events. Selective COX-2 inhibitors have also been associated with gastrointestinal symptoms, primarily as a result of the inhibition of mucosal protective prostaglandins [3,4,16]. The prevalence of gastrointestinal events was greater in the celecoxib groups of the Arber Study. Additionally, renal disease or hypertension was significantly higher in the celecoxib group of the Arber study, and also in one of the two celecoxib groups of the Bertagnolli study [7]. In our study, we did not include adverse events as outcomes. During follow-up, we found that overall, there was a greater number of cancer patients using COX-2 inhibitors, however the average number was lower than those seen for the control group. This is possibly because of adverse events, therefore administration of medication has to be discontinued. We investigated patients without any occurrence of myocardial infarction or congestive heart failure and peptic ulcer disease. It was found that the estimates of risk reduction were not largely different in patients without cardiovascular events. Therefore, the occurrence of cardiovascular events might not have effect on the association of COX-2 inhibitors and CRC. In terms of peptic ulcer disease, greater increases in the risk reduction were observed in patients with occurrence of peptic ulcer disease. However, in this study we did not have enough sample size to provide sufficient statistical evidence.

We have included both refecoxib and celecoxib in the analysis. Since rofecoxib was withdrawn in 2004, the study results may not directly reflect the effect of the current available COX-2 inhibitor (celecoxib).

The ICD-9 codes of 140–208 are sometimes provided by the Taiwan NHI program for cancer-screening purposes. The result of this is that the incidences of cancer can be greatly overestimated. In our study, CRC patients were identified by linking their encrypted personal identification number to the Registry for Catastrophic Illness patients with ICD9 code 153–154. For patients to be in the Registry for Catastrophic Illness, their medical records need to be reviewed so that they can qualify for 100% reimbursement of disease-related medical expenses. Our database comprised approximately 90% of the Taiwan cancer incidence registry. A possible reason why patients might not appear in the catastrophic illness registry of the NHI program may include short period between diagnosis of CRC and death. These identified patients were excluded from the study. Therefore, we believe that 90% of CRC patients in the Catastrophic Illness Registry was a reasonable representation of CRC patients in Taiwan.

Our study also has limitations on some key confounders of CRC, including familial adenomatous polyposis [17], calcium [18], folate, methionine and alcohol intake[19] as well as exercise, obesity and smoking habit [7]. These factors were not recorded in the NHI database, and might reduce the estimates of risk reduction, if they were included in regression analyses. For a given participant, the usage of selective COX-2 inhibitors might have been affected by co-prescriptions of other NSAIDs. To adjust for this situation, the conditional logistic regressions were also conducted with two MPR covariates for tNSAIDs and preferential COX-2.

The database used did not include information for over-the-counter use. Hence, some underestimation of the NSAIDs used may have occurred. Because the Taiwan NHI program provides comprehensive medication coverage, any drug use not recorded in the database would be limited to short-term relief of symptoms, and the effect on the study results is therefore limited.

It has been speculated that the development of an adenoma into CRC may take as long as 10–15 years [7]. Given that outcomes can only be assessed by colonoscopy, there may be some false negatives in the control group. The control group included people without CRC or other cancers before and after the index dates. This was to prevent possible misclassification due to late diagnosis of cancers. In the sensitivity analysis, we also investigated patients without any occurrence of colon and rectal polyps. It was found that the estimates of risk reduction were not largely different from the main analysis. Therefore, the effect on the association of COX-2 inhibitors and CRC might be limited.

## Conclusion

Few studies have focused on the chemopreventive effects of selective COX-2 inhibitors on CRC in the general population. The results support the chemopreventive role of selective COX-2 inhibitors in CRC. Risk reduction occurred after 6 months, 3 years and 5 years of continual use of the drugs. Additionally, the frequencies of use for COX-2 inhibitors from 1–5 years may be as low as 10% of MPRs to achieve at least 10% risk reduction with respect to developing CRC. Given limited reports from individuals with no increased risk of CRC, our results provide information in the general population.

## Abbreviations

COX-2: Cyclooxygenase-2; CRC: Colorectal cancer; NHIRD: Health Insurance Research Database; NSAIDs: Non-steroidal anti-inflammatory drugs; MPR: Medication possession ratio; tNSAID: Traditional NSAID.

## Competing interests

The authors declare that they have no competing interests.

## Authors’ contributions

YHY performed statistical analyses and drafted the manuscript. YHKY and CLL participated in the study design, and helped to draft the manuscript. PSH and YCK provided important inputs to the manuscript. All authors read and approved the final manuscript.

## Pre-publication history

The pre-publication history for this paper can be accessed here:

http://www.biomedcentral.com/1471-2407/12/582/prepub
